# Acculturation and eating disorders: a systematic review

**DOI:** 10.1007/s40519-023-01563-2

**Published:** 2023-04-19

**Authors:** Sarah Song, Casey M. Stern, Tzivia Deitsch, Margaret Sala

**Affiliations:** Ferkauf Graduate School of Psychology, Yeshiva University, Bronx, NY USA

**Keywords:** Acculturation, Acculturative stress, Culture change, Eating disorders, Disordered eating

## Abstract

**Purpose:**

Acculturation, or the dual process of cultural change that takes place due to the interaction between two or more cultural identities, may contribute to the susceptibility of developing an eating disorder (ED). We conducted a systematic review exploring the relationship between acculturation-related constructs and ED pathology.

**Methods:**

We searched the PsychINFO and Pubmed/Medline databases up to December 2022. Inclusion criteria were: (1) having a measure of acculturation or related constructs; (2) having a measure of ED symptoms; and (3) experiencing cultural change to a different culture with Western ideals. 22 articles were included in the review. Outcome data were synthesized by narrative synthesis.

**Results:**

There was variability in the definition and measure of acculturation in the literature. Overall, acculturation, culture change, acculturative stress, and intergenerational conflict were associated with ED behavioral and/or cognitive symptoms. However, the nature of the specific associations differed depending on the specific acculturation constructs and ED cognitions and behaviors measured. Furthermore, cultural factors (e.g., in-group vs. out-group preferences, generational status, ethnic group, gender) impacted the relationship between acculturation and ED pathology.

**Discussion:**

Overall, this review highlights the need for more precise definitions of the different domains of acculturation and a more nuanced understanding of the specific relationship between various acculturation domains and specific ED cognitions and behaviors. Most of the studies were conducted in undergraduate women and in Hispanic/Latino samples, limiting generalizability of results.

**Level of evidence:**

Level V, Opinions of respected authorities, based on descriptive studies, narrative reviews, clinical experience, or reports of expert committees.

## Understanding the relationship between acculturation and eating disorders

The three main categories of eating disorders (EDs) are anorexia nervosa (AN), bulimia nervosa (BN), and binge eating disorder (BED). The lifetime prevalence of EDs is approximately 0.16% for AN, 0.63% for BN, and 1.6% for BED [1]. EDs are a serious public health concern due to their numerous physical and social consequences [2]. Although EDs were initially conceptualized to only impact Western countries, more recent literature suggests that a variety of racial and ethnic populations struggle with EDs in the United States and globally [3]. Culture change and acculturation to Western culture may contribute to the development of EDs [4, 5].

### Acculturation

*Acculturation* has been defined as the dual process of cultural and psychological change that takes place as a result of contact between two or more cultural groups and their members [6]. An individual who is “more accultured” is considered to be more adjusted or integrated to Western culture. The process of culture change and acculturation may contribute to the development of EDs. Western culture places paramount importance on physical appearance and a thin body, increasing pressure to attain such thin body through excessive dieting and potential engagement in ED behaviors. Western cultural values stand in contrast to values from other cultures, which often place a lower value on physical appearance and/or include alternative beauty ideals (e.g., less emphasis on thinness, a larger or curvy body ideal) [7]. Acculturation to Western culture is often accompanied by cultural messages related to Western beauty standards (e.g., the thin-ideal), which may contribute to engaging in EDs [8]. Indeed, research has shown that culture change has been associated with increases in ED symptoms [9].

There are differences in how individuals choose to adjust to new cultures that may influence susceptibility to EDs. Specifically, research has shown that individuals may cope with culture change through four different acculturation strategies: (1) marginalization, or rejecting both original and new cultural identities; (2) separation, or preserving original cultural identity; (3) integration, or maintaining original cultural identity while adopting new cultural identity; and (4) assimilation, or only adopting new cultural identity [10]. Previous literature suggests that maintenance of both cultural identities (i.e., integration) is linked with better overall outcomes than maintenance of only one culture (i.e., assimilation or separation) [14]. Notably, marginalization and separation strategies of acculturation are associated with an increased vulnerability for disordered eating. This is the case because both marginalization and separation lack integration into the new culture, and disordered eating may be the result of dysfunctional mechanisms to cope with the internal conflicts that arise with cultural adjustment [10].

### Acculturative stress

Acculturation may also contribute to the development of EDs due to the high amounts of stress experienced in the process of culture change [9]. Those who experience acculturation are exposed to the likelihood of developing *acculturative stress*, or psychological/physical/social difficulties related to adjusting from one culture to another. Although acculturative stress is a core component of acculturation, there is significant overlap between acculturation and acculturative stress in the literature [11]. Stressors related to acculturation include learning a new language, adjusting to different societal norms, and navigating familial disagreements and different levels of acculturation between generations within the family unit [12]. These stressors may subsequently lead to health concerns and various psychological difficulties (e.g., depression, suicide) [13, 14], as well as using disordered eating behaviors as a means of coping [15]. Previous research has linked acculturative stress with psychological distress (e.g., lower self-esteem, depressive symptoms), as well as ED symptoms [4, 5].

### Intergenerational conflict

In addition to being associated with acculturative stress, acculturation is also often associated with *intergenerational conflict*, which is discord in the family unit due to an acculturation gap among the family members [16]. Compared to children of immigrant families, parents are often less acculturated to dominant culture [17] due to their tendencies to maintain their original cultural norms (e.g., maintaining native language and traditional cultural values) [18]. One study suggested that while acculturation does not predict psychological distress, familial conflict due to cultural differences does [19]. In other words, differences in familial reactions to acculturation may be a specific aspect of acculturation that contributes to increased psychological distress [19].

### Previous reviews

Doris et al. [20] conducted a systematic review investigating the relationship between ED symptoms (i.e., those seen in AN and BN) and acculturation to the Western culture. More specifically, they examined whether exposure to the Western culture via acculturation and immigration contributed to the development of ED symptomology. They reviewed 25 studies and found mixed results. Some studies noted that acculturation was related to higher vulnerability of developing an ED, whereas others indicated that acculturation was associated with lower ED symptomology. They noted that differences in the sample recruited and differences in how acculturation was defined, conceptualized, and measured contributed to the discrepancies in their findings. One major limitation of the Doris et al. review [20] was the exclusion of the relationship between acculturation and binge eating symptoms. More recently, Warren and Akoury [8] conducted a systematic review exploring the relationship between acculturation-related constructs, thin-ideal internalization, and eating pathology. They found positive associations between thin-ideal internalization, acculturative stress, and eating pathology. However, findings on the relationship between other aspects of acculturation and eating pathology were mixed. Of note, Warren and Akoury limited their review to studies that included measures of thin-ideal internalization, and therefore excluded studies that only measured acculturation and eating pathology.

### Current study

The purpose of this literature review is to expand upon previous reviews [8, 20] by examining how the association between acculturation and ED pathology may differ based on the specific acculturation construct (e.g., acculturation vs. culture change vs. acculturative stress vs. intergenerational conflict) and ED cognition/behavior measured (e.g., restricting vs. binge eating). In addition, in contrast to previous reviews [8, 20], we included articles focused on binge eating and BED in our search, and did not limit our search to articles including measures of thin-ideal internalization.

## Methods

This review was conducted in accordance with the Preferred Reporting Items for Systematic Reviews and Meta-Analyses (PRISMA) guidelines [21].

### Literature search

**Fig. 1:**
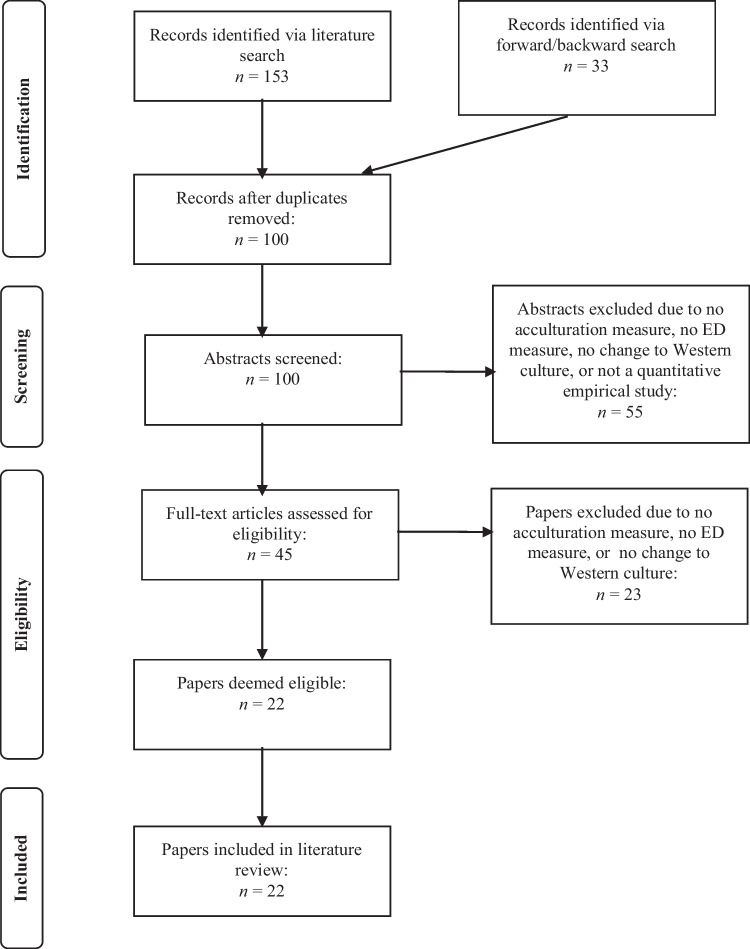
PRISMA flow diagram

We identified relevant articles using the PsychINFO and Pubmed/Medline databases. Search keywords included “accultur*” or “*culture change”* along with combinations of “*eating disorder”* or “anorex*” or “bulim*” or “bing*” or “*disordered eating*.” We determined phrases for search criteria based on similar previous reviews. The search was limited to English peer-reviewed journals written after 2015 because this systematic review is an update of the review conducted by Doris et al. [20]. We did not consider grey literature (e.g., dissertations) to maintain the quality of included studies. The search was conducted by the first three authors (SS, CS, TD), including articles up to November 2022. The study was not pre-registered, as this was a master’s thesis, and we were not sure if we would pursue publication at the initial research stages. The initial search generated 186 research articles. After eliminating abstracts that were duplicates, a total of 100 article abstracts were screened. 45 full texts were assessed for eligibility, and 22 papers were ultimately included in the review. See Fig. 1 for the PRISMA flow diagram.

Informed consent to participate in each respective study cited in this review was obtained from all participants. In the case of minors, informed consent was obtained from a parent or legally authorized representative and assent was obtained from the participant.

### Screening

#### Inclusion criteria

Studies were included if they had: (1) a measure of acculturation or related constructs (e.g., ethnic identity, acculturative stress, intergenerational conflict, culture change); (2) a measure of ED symptoms; and (3) participants were experiencing cultural change to a different culture with Western ideals (e.g., White, US, Europe), regardless of generation status. We omitted case studies, review papers, and studies that examined within-culture acculturation or adjusting to similar cultures (e.g., internal migration from rural to urban settings within one country), because the focus of this review was to explore whether migration from non-Western (any culture outside of the predominant White Western culture) to Western culture is associated with ED pathology. Of note, given the heterogeneity in acculturation measures and construct definitions, we were guided by previous systematic reviews on acculturation (Doris et al. 2015) when deciding whether a specific measure was a measure of acculturation or related construct.

### Data review

After identifying the included articles, specific data were collected from each study. We used a data extraction template that collected information regarding authors, number of participants, population (e.g., Asian American female college students, community sample of Hispanic/Latino men, and Emirati female college students), countries represented, percentage of men, mean age, study design, ED measure(s), acculturation measure, study objective, and results. Data were coded by the first and second authors. All disagreements were resolved by the last author.

## Results

We identified 22 articles that examined the relationship between acculturation and ED pathology (see Fig. 1). See Table 1 for pertinent study characteristics and summarized results. After reviewing the articles, the results were grouped into three categories based on the relevant results from those articles: (1) the associations between acculturation and ED behaviors; (2) the associations between acculturation and ED cognitions; and (3) differences in cultural factors (i.e., in-group vs. out-group preferences, generational status, ethnic group, gender).

**Table 1:** Summary of included studies

Authors (year)	*N*	Population	Countries/ethnicities represented	% Men	Mean age	Design	ED measures	Acc. measure	Results summary
Akoury et al. (2019)	430	Asian American female college students	Filipino, Chinese, Japanese, Vietnamese, Korean, Other Asian American, South Asian, Thai, Taiwanese, Cambodian, Hawaiian/Pacific Islander, Laotian	0%	20.64	CS	EDE-Q*	SATAQ-4 MEIM BIIS SAFE	**Significan**t: Acculturative stress was positively associated with pressures for thinness, thin-ideal internalization, and disordered eating **Non-significant**: Ethnic identity was not correlated with disordered eating **Mediators**: Thin-ideal internalization partially mediated relationships between pressures to be thin and disordered eating. Acculturative stress fully accounted for the relationship between biculturalism and disordered eating
Claudat et al. (2015)	638	Asian American and Latina female college students	Not available	0%	19.88	CS	EDE-Q*	SAFE	**Significant**: Higher levels of acculturative stress were associated with higher levels of eating pathology in both Asian American and Latina students **Mediators**: Self-esteem was an indirect mediator of the relationship between acculturative stress and eating pathology
Cordero et al. (2022)	1,463	Hispanic/ Latinx children in Chicago, IL; Miami, FL; Bronx, NY; San Diego, CA	Mexican, Central American, South American, Cuban, Puerto Rican, Dominican, other/more than one background	42.8%	13 for adolescents, 11 for children	CS	SOL Youth disordered eating questionnaire*	SATAQ* AHIMSA	**Significant**: Acculturative stress was associated with dieting ≥ 5 times a year, overeating, and compensatory behaviors **Not significant**: Acculturation did not significantly predict disordered eating behaviors
Han (2020)	244	Asian and Asian American female college students	Chinese, South Korean, Taiwanese, Indian, Pakistani, Japanese, Hong Kongers/Hongkongese, Chinese American, Korean American, Indian American, Vietnamese American, Filipino American, Taiwanese American, Japanese American, Thai American, Malaysian American	0%	21.66	CS	BES* DEBQ (Restrained Eating subscale)	AAFCS AAVSM	**Significant**: There was a positive correlation between intergenerational conflict and binge eating behaviors as well as restricting behaviors **Mediators**: Psychological need thwarting and anxious parental attachment indirectly mediated the relationship between intergenerational conflict and binge eating
Higgins & Bardone-Cone (2017)	119	Female bicultural Latina participants who predominantly have a history of binge eating disorder and/or bulimia nervosa	Not available	0%	20.12	CS	EDE-Q*	SAFE	**Significant**: Acculturative stress was positively associated with binge eating **Mediators**: Negative affect indirectly (but not directly) mediated the correlation between acculturative stress and binge eating
Johanesen et al. (2022)	285	Latino adolescents	Not available	42%	15.9	CS	EDDS DEBQ	AHIMSA	**Not significant**: Acculturation was not associated with binge eating, restrained, emotional, or external eating **Mediators**: Combined emotional, external, and restrained eating indirectly mediated the relationship between acculturation and binge eating (but not individually)
Johnson et al. (2022)	2554	Latinos, Asian Americans age 18 +	Cuban, Puerto Rican, Mexican	44.2%	40.6	CS	CIDI	HIS MAPSS	**Not significant**: Acculturative stress was not associated with binge eating
Kwan et al. (2018)	187	Ethnic minority undergraduate students (Asian, Pacific Islander, African American, Hispanic/ Latino, American Indian, Alaska Native)	Chinese, Nepalese, South Korean, Indian, Canadian, Saudi Arabian	41.2%	20.45	CS	EDE-Q* EDI*	SAFE	**Significant**: Higher acculturative stress was linked to more eating concern, shape concern, weight concern, drive for thinness, and bulimic symptoms **Non-significant**: Acculturative stress was not linked to body dissatisfaction or restraint **Moderation**: Gender moderated the effect of acculturative stress on drive for muscularity. Acculturative stress was only linked with drive for muscularity in women
Kolodziejczk (2015)	205	Community sample of predominately Hispanic overweight and obese adolescents in California	Not available	57.6%	12.9	CS	EDI*	SASH	**Non-significant**: Acculturation did not moderate the relationship between BMI and HRQOL
Lawson et al. (2019)	444	Hispanic adults seeking bariatric surgery	Not available	16.5%	37.3 (11.0)	CS	YFAS 2.0	SASH	**Significant**: Acculturation was positively associated with food addiction symptoms and with BMI. Within each of the English-speaking and Spanish-speaking sample, those who endorsed lower acculturation had lower BMI. Acculturation significantly predicted food addiction symptoms
Marquez and Benitez (2021)	59 dyads	Mexican–American mothers and adult daughters	Mexican, Mexican–American	0%	25.1 for daughters, 49.8 for mothers	CS	EAT-26*	BAS	**Significant**: Daughter acculturation was positively associated with maternal bulimic symptoms **Not significant**: Daughter acculturation was not significantly associated with daughter BMI, body size satisfaction, bulimia symptoms, or dieting, nor with maternal negative or positive weight-related messages, or maternal dieting. Acculturation did not predict any bulimic or dieting behaviors in daughters
Negi et al. (2022)	112	Undergraduate women from South and Southeast Asian descent	Indian, Pakistani	0%	23.1	CS	EAT-26*	SL-ASIA	**Not significant**: Acculturation was not associated with disordered eating **Mediators**: Thin-ideal internalization fully accounted for the path between acculturation and disordered eating
Neyland and Bardone-Cone (2019)	43	Latina female community sample	Not available	0%	20.58	CS	EDDS*	SMAS	**Significant**: Perceived helpfulness of treatment was positively correlated with acculturation to White dominant society (i.e., dominant society immersion) **Not significant**: There was no significant relationship between acculturation and total number of treatments or mental health treatment stigma tolerance
Saunders et al. (2015)	1339	Ethnically diverse co-ed college student sample (82% Hispanic)	Cuban, South American, Central American, Puerto Rican, Dominican, Mexican, Spanish	35.9%	21	CS	EAT-26*	SASH	**Not significant**: No significant relationship between acculturation and overall eating disorder risk
Shekriladze et al. (2019)	506	Georgian women living in either the UK/USA or Georgia	Georgian	0%	41	CS	EDE-Q*	EAAM	**Significant**: Marginalization and separation were positively correlated with eating concern, shape concern, weight concern, and global scores. There was positive relationship between all acculturation strategies and restricting behaviors. Integration was negatively associated with eating concern and shape concerns **Non-significant**: There was no significant correlation between assimilation and eating pathology, or between integration and weight concern or global scores
Swami (2016)	98	Female students from Malaysia starting higher education in the UK	Malaysian	0%	18.35	Mix: L and CS	EDI	Sociocultural Adjustment measure Cultural Distance measure	**Significant**: At time 2 (four months after the migration), there was a higher risk for eating disorder pathology compared to time 1 (two months prior to the migration). Sociocultural adjustment was associated with higher scores on drive for thinness, body dissatisfaction, and bulimia symptoms. Cultural distance was associated with higher drive for thinness **Non-significant**: There was no significant relationship between cultural distance and body dissatisfaction or bulimic symptoms
Thomas et al. (2016)	94	Arab female college students	Arab/Emirati	0%	21.47	CS	EAT-26*	Westernization Survey MIIS	**Significant**: Implicit in-group preference was negatively associated with overall eating disorder symptomology. Out-group identification had significantly higher eating disorder symptoms compared to in-group preference **Non-significant**: Explicit in-group evaluations and Western acculturation were not linked to overall eating disorder symptoms
Thomas et al. (2017)	209	Emirati female college students	Arab/Emirati	0%	25.36	CS	EAT-26*	Westernization Survey	**Significant**: Implicit out-group preferences and Western acculturation were positively associated with eating pathology. Participants considered at-risk for eating disorders endorsed out-group preferences significantly more than those who were not at risk. Participants considered at-risk also demonstrated significantly greater Western acculturation than those not at risk
Wang et al. (2022)	477	Asian undergraduate students	Chinese, Vietnamese, Filipino, Korean, Indian, Japanese	78%	20.35	CS	TFEQ	RASI	**Significant**: Higher acculturation was associated with higher disinhibition **Mediators/Moderators**: Depressive symptoms showed a partial indirect effect for the association between acculturation and disinhibition. This indirect correlation was stronger among males than females
Williamson et al. (2019)	271	Community sample of Hispanic/ Latino men	Mexican, Puerto Rican, Other Hispanic, Spanish, Dominican, Cuban, Colombian, Honduran, Ecuadorian	100%	23.89	CS	MBAS-R EDE-Q	SASH	**Significant**: Acculturation was negatively associated with loss-of-control eating **Non-significant**: Acculturation did not moderate the relationship between body image concerns and muscularity concerns
Wu et al. (2020)	166	Community sample of higher-weight Asian Americans living in North Carolina	Not available	55.4%	45.7	CS	BES*	SL-ASIA	**Significant**: Acculturation was positively associated with experienced weight stigma and with binge eating **Non-significant**: Acculturation did not moderate the correlation between weight stigma and binge eating
Zhou et al. (2022)	245	Asian-American college students	Chinese, Taiwanese, Vietnamese, Korean, Filipino, Japanese, South Asian, Indian, Nepalese	33.3%	20.36 (1.58)	CS	OBCS EDE-Q*	AAMAS SATAQ-4	**Significant**: Adoption of American culture was positively associated with muscular body ideal internalization **Not significant**: Adoption of American culture was not significantly associated with thin body ideal, body surveillance, body shame, disordered eating, or log-transformed BMI. No main effect of American culture on any eating- or body image-related outcome measures **Moderation**: There was an interaction effect such that those with higher levels of both Asian and American culture had the greatest risk of muscular body ideal internalization

*ED* eating disorder; *Acc*. acculturation; *CS* cross-section; *EDE-Q* Eating Disorder Examination Questionnaire; *SATAQ-4* Sociocultural Attitudes Toward Appearance Questionnaire -4; *MEIM* Multigroup Ethnic Identity Measure, *BIIS* Bicultural Identity Integration Scale; *SAFE* Societal, Attitudinal, Familial, and Environmental Acculturative Stress Scale; *AHIMSA* Acculturation, Habits and Interests Multicultural Scale for Adolescents; *BES* Binge Eating Scale; *DEBQ* Dutch Eating Behavior Questionnaire; *AAFCS* Asian American Family Conflict Scale, *AAVSM* Asian American Value Multidimensional Scale; *EDDS* Eating Disorder Diagnostic Scale; *CIDI* Composite International Diagnostic Inventory; *HIS* Hispanic Stress Inventory; *MAPSS* Mexican-American Prevalence and Services Survey; *EDI* Eating Disorder Inventory; *SASH* Short Acculturation Scale for Hispanics; *BMI* Body Mass Index; *HRQOL* Health-Related Qualirt Of Life; *YFAS 2.0* Yale Food Addiction Scale 2.0; *EAT-26* Eating Attitudes Test; *SL-ASIA* Suinn–Lew Asian Self Identity Acculturation Scale; *SMAS* Stephenson Multigroup Acculturation Scale; *EAAM* East Asian Acculturation Measure; *L* Longitudinal; *MIIS* Multicomponent In-group Identification Scale; *TFEQ* Three Factor Eating Questionnaire; *RASI* Riverside Acculturative Stress Inventory; *MBAS-R* Revised Male Body Image Attitudes Scale; *AAMAS* Asian American Multidimensional Acculturation Scale; *OBCS *Objectified Body Consciousness Scale

### The association between acculturation and ED behaviors

#### Bulimic symptoms

Overall, culture change and higher levels of acculturative stress are associated with higher levels of bulimic symptoms (i.e., episodes of binge eating and purging) [9, 22]. In contrast, there appears to be no significant relationship between acculturation and bulimic symptoms. Specifically, Swami [9] identified a relationship between culture change (i.e., migration) and bulimic symptoms in Malaysian students migrating to the United Kingdom, with bulimic symptoms increasing after migration compared to before migration. Two studies examined the relationship between acculturative stress and bulimic symptoms. Findings from both studies suggest a significant relationship such that higher acculturative stress was associated with higher bulimic symptoms [22, 23]. In contrast, there appear to be null relationships between acculturation and compensatory behaviors or bulimic symptoms [24].

#### Binge eating and loss-of-control eating

Some research suggests that higher levels of acculturative stress and intergenerational conflict are associated with higher levels of binge eating symptoms (i.e., consuming large quantities of food in a short amount of time with a sense of loss of control) as well as related constructs (e.g., disinhibited eating) [4, 25–27]. Specifically, three studies examined the relationship between acculturative stress and binge eating behaviors. Results from two of these studies suggest a significant relationship such that acculturative stress was associated with higher binge eating or overeating [4, 23]. However, results from one study suggest no significant relationship between acculturative stress and binge eating [28]. Han [25] investigated the influence of intergenerational conflict on eating pathology behaviors, including binge eating, in Asian and Asian American college students. Han [25] found that intergenerational conflict was associated with binge eating through thwarted psychological needs. Intergenerational conflict was attributed to the differences in the degree of acculturation between parents and their children (e.g., parents being less likely than their children to assimilate into US cultural values and being more likely to maintain traditional values).

The relationship between acculturation and binge eating is less clear. Six studies examined the relationship between acculturation and binge eating behaviors. Of these, three suggest a significant relationship such that acculturation was associated with higher disinhibition, binge eating, or food addiction symptoms [26, 29, 30]. On the other hand, results from one study suggest a negative relationship such that higher acculturation was associated with lower loss-of-control eating [27]. Results from two studies suggest no relationship between acculturation and overeating or binge eating [23, 31].

Results from two studies suggest that depression and negative affect may indirectly mediate the association between acculturative stress and binge eating/disinhibited eating [4, 26]. Emotional, external, and restrained eating may mediate the association between acculturation and binge eating [31].

#### Restricting

Overall, culture change and intergenerational conflict were associated with higher levels of restricting behaviors (i.e., placing restrictions on quantity and type of food consumed, such as via calorie counting, meal skipping, and following rules) [10, 25]. Specifically, Shekriladze et al. [10] explored the relationship between different strategies of acculturation (i.e., integration, assimilation, separation, marginalization) and eating pathology, including restricting behavior, in a sample of Georgian women. They found that dietary restriction increased upon migration, regardless of the acculturation strategies used. Han [25] reported that intergenerational conflict was predictive of restricting behaviors through thwarted psychological need. However, the size of the effect was smaller than that of the relationship between intergenerational conflict and binge eating.

The relationship between acculturative stress and restricting was less clear. Two studies examined the relationship between acculturative stress and restrictive eating, restraint, or dieting. Results from one of these studies suggest a significant, positive association such that acculturative stress was associated with dieting behavior [23]. Results from the other study suggest no significant relationship between acculturative stress and restraint [22]. In contrast, acculturation does not appear to be significantly associated with restricting behaviors. Specifically, three studies examined the relationship between acculturation and restrictive eating, restraint, or dieting. All three found no significant relationship between acculturation and dieting or restrained eating [23, 24, 31].

### The association between acculturation and ED cognitions

#### Drive for thinness

Results from several studies suggest that culture change and higher levels of acculturative stress are associated with higher levels of drive for thinness, or an excessive concern with dieting and weight [9, 22, 32]. Specifically, Swami [9] explored the relationship between culture change (i.e., migration) and ED cognitions, including drive for thinness, in female students from Malaysia that moved to the United Kingdom. They found a significant increase in drive for thinness scores after migration, compared to before migration. Two studies examined the relationship between acculturative stress and thin-ideal internalization or drive/pressure for thinness. Both studies found a significant relationship such that acculturative stress was associated with higher drive for thinness, thin-ideal internalization, or pressure for thinness [22, 32]. In contrast, results from one study suggest a lack of significant relationship between acculturation and thin-ideal internalization [33].

#### Eating, shape, and weight concern

Research suggests that higher levels of acculturative stress [22] and the specific acculturation strategies of marginalization and separation [10] are associated with higher levels of eating concerns, shape concerns, and weight concerns (i.e., preoccupation with eating, shape, and/or weight). Specifically, one study examined the relationship between acculturative stress and shape or weight concerns, and there was a significant relationship such that acculturative stress was associated with both higher shape concerns and higher weight concerns [22]. Shekriladze et al. [10] investigated the relationship between different acculturation strategies (i.e., marginalization, separation, integration, and assimilation) and eating pathology in Georgian women living in the UK, US, or Georgia. Their findings indicated that more engagement of marginalization and separation were related to higher eating concerns, shape concerns, and weight concerns. In contrast, integration was associated with lower levels of eating and shape concerns.

#### Body dissatisfaction

Overall, it appears that while culture change is associated with higher body dissatisfaction (i.e., not being satisfied with one’s body), acculturation and acculturative stress are not significantly associated with body dissatisfaction. Specifically, Swami [9] found that that body dissatisfaction increased after migration, compared to before migration. In contrast, acculturation and acculturative stress do not appear to be associated with body dissatisfaction. Two studies examined the relationship between acculturation and body dissatisfaction or shame. Both found no relationship [24, 33]. Kwan et al. [22] found that acculturative stress was not associated with body dissatisfaction in a sample of ethnic minority undergraduate students.

### Differences in cultural factors

#### In-group vs out-group preferences

Research suggests that out-group preferences, or being more acculturated to Western values than to non-Western values, are associated with higher levels of ED symptoms [14]. Specifically, Thomas et al. [14] investigated how implicit and explicit measures of in-group preferences vs. out-group preferences in Arab female college students were related to ED symptoms. Out-group identification was conceptualized to be acculturated to Western values while in-group preferences were aligned with Emirati cultural behaviors. They assessed for explicit in-group preferences through a self-report measure, and for implicit in-group preferences using an affective priming task comparing response times to common Emirati first names (e.g., Shamsa) vs. common American first names (e.g., Emma). They found that higher implicit in-group preferences were related to lower ED symptomology. In contrast, there was no association between explicit in-group evaluations and ED symptoms. They also reported those who identified with out-group preferences endorsed higher ED symptoms. Overall, findings from this study suggest that maintaining traditional cultural values was a protective factor against ED pathology. Consistent with these findings, Thomas et al. [34] revealed that Emirati female undergraduate students at risk for EDs were more likely to endorse out-group preferences (e.g., engaging in more Western behaviors such as speaking English or watching Western TV shows) and Western acculturation compared to those who were not at risk. In addition, they found a positive correlation between implicit out-group preferences and eating pathology.

#### Generational status differences

Tsong and Smart [35] found that differences in generational status were associated with ED pathology in a community sample of Asian American women. They defined first-generation status as being born in another country and moving to the United States after the age of 12 years; second-generation status as being born in the US and having parents born in a different country; and third-generation status as being born in the US and having parents born in the US. They found that second-generation status was associated with the highest overall eating pathology. That is, second-generation Asian American women endorsed the most disordered eating tendencies compared to first-generation and third-generation Asian American women.

#### Ethnic group differences

Other researchers compared how the relationship between acculturation and ED symptomology may differ among diverse ethnic groups. Claudat et al. [12] found that higher acculturative stress was associated with higher levels of eating pathology in both Asian American and Latina students. Notably, Asian American women endorsed more acculturative stress than Latina women in this sample. Saunders et al. [36] revealed that there was no significant relationship between acculturation and overall ED symptomology in either non-Hispanic or Hispanic individuals [36]. Furthermore, the Hispanic and non-Hispanic individuals did not differ in regard to their engagement in restrictive, bulimic, and binge eating behaviors. Interestingly, one study indicated a positive influence of acculturation in that acculturation to dominant White culture was positively correlated with perceived helpfulness of ED treatment in a sample of Latina females (i.e., BED and BN) [37].

#### Gender differences

Kwan et al. [22] found that gender moderated the relationship between acculturative stress and drive for muscularity, where acculturative stress was only linked with drive for muscularity in women. However, gender did not moderate the relationship between acculturative stress and other ED constructs (e.g., restriction, eating/weight/shape concern, drive for thinness, bulimia, body dissatisfaction). Recent research suggests that mediators may vary by gender. For example, Wang et al. (2022) [26] found that that the indirect association between acculturative stress and eating disinhibition via depression symptoms was stronger for Asian men than women.

## Discussion

This systematic review aimed to synthesize the literature from 2015 to 2022 on the association between acculturation to Western culture and ED pathology. In addition to acculturation, the studies we included examined various constructs related to acculturation, including intergenerational conflict, acculturative stress, culture change and the specific acculturation strategies used. Overall, acculturation, culture change, acculturative stress, and intergenerational conflict were associated with ED behavioral and/or cognitive symptoms. However, the nature of the specific associations differed depending on the specific acculturation constructs and ED behaviors/cognitions measured. Furthermore, various cultural factors (i.e., in-group vs. out-group preferences, generational status, ethnic group, gender) impacted the relationship between acculturation and ED pathology. We add to previous reviews on the topic [8, 20] by identifying how acculturation and related constructs are associated with specific ED behaviors and cognitions, as well as moderators of these relationships.

Acculturation, culture change, acculturative stress, and intergenerational conflict were associated with several different ED behaviors, but the nature of the relationships differed depending on the specific acculturation constructs and ED behaviors measured. Specifically, culture change was associated with higher levels of bulimic symptoms [9] and restrictive behaviors [10]. Individuals may shift to being influenced by Western thin ideals when changing cultures instead of non-Western beauty ideals with a lower emphasis on the value of physical appearance and thinness, resulting in restrictive and bulimic behaviors to manipulate their body size. Higher levels of acculturative stress were associated with more engagement in bulimic symptoms [22, 23] and binge eating/loss-of-control eating [4, 23]. This may be because binge eating and bulimic behaviors may function as a way to escape negative affect or aversive self-awareness [4, 26]. Finally, higher levels of intergenerational conflict were associated with higher levels of binge eating and restrictive behaviors [25]. This may be because ED behaviors may function to compensate for differences in levels of acculturation from parents. Overall, these findings highlight that the nature of the relationship between acculturation and its domains with ED behaviors varies depending on the specific constructs examined, and highlight the importance of distinguishing different constructs related to acculturation (e.g., culture change vs. acculturative stress vs. acculturation) in order to understand the specific relationships between acculturation and specific ED behaviors.

Culture change was associated with various ED cognitions, including higher levels of drive for thinness and body dissatisfaction [9]. Acculturative stress was associated with higher levels of drive for thinness, and weight/eating/shape concerns [22, 32], but was not associated with body dissatisfaction [22]. The specific acculturation strategies used were also related to ED cognitions. Specifically, whereas higher marginalization and separation were associated with higher levels of ED cognitions (e.g., weight concern, shape concern, eating concern), higher integration was associated with lower levels of ED cognitions [10]. This may be because marginalization and separation both reject new cultural identity in the process of acculturation, and this lack of integration may contribute to various ED cognitions. In contrast, integration manages both the original and new cultural identity, which may reduce the stress associated with acculturation. These findings are consistent with literature in other fields in which preferences for marginalization and separation were associated with more identity confusion, anxiety, aggression, and anger [38], whereas integration was associated with psychological benefits (e.g., higher life satisfaction, higher self-esteem, lower psychological distress) [39].

Finally, there appear to be differences in the relationship between acculturation and ED pathology in the context of cultural factors. First, researchers reported that identification with out-group preferences was associated with higher ED symptoms [14]. This may be because Westernization may normalize weight loss behaviors and ED psychopathology, and because identifying with the Western culture may be related to accepting its thinness and beauty ideals. Second, second-generation immigrants are particularly susceptible to ED pathology. For example, second-generation Asian American women endorsed the highest overall eating pathology compared to first and third-generation Asian American women [35]. This finding is consistent with previous literature which found the highest depression rates in second-generation Mexican-American females [40], and overall poorer mental health [41]. Second-generation individuals may have a unique experience with acculturation that may play a role in the development of eating pathology and higher levels of biculturative stress (i.e., the stress that develops from managing both original and new cultural identities) compared to first- and third-generation immigrants. Future research may consider empirically examining why there may be differences in ED pathology among first-generation, second-generation, and third-generation immigrants. Third, limited research has investigated how the relationships between acculturation and ED pathology may differ among different cultural groups. Overall, it appears that acculturation and/or its domains are associated with ED pathology across various cultural groups (e.g., Asian Americans, Latinos), but future research is needed to understand specific differences that may exist. Finally, acculturation may be more strongly associated with some types of ED pathology in women than in men. For example, one study found that there was a relationship between acculturative stress and drive for muscularity in women but not men [22]. This may be due to multiple minority memberships, where women’s double minority status (i.e., ethnic minority and gender identities) may have contributed to this relationship as a way of adjusting to the mainstream culture. However, it was not clear why gender only moderated the relationship between acculturative stress and drive for muscularity, but not other ED constructs measured (e.g., restriction, eating/weight/shape concern, drive for thinness, bulimia, body dissatisfaction), and future research will need to investigate why gender moderates the relationship between acculturative stress and only some ED behaviors.

### Strengths and limitations

Strengths of this review included an updated investigation of this very important and under-researched topic, a synthesis of the complexities in the literature, and a more detailed analysis about which specific ED constructs acculturation may be related to as compared to previous reviews. There are a few limitations to consider when evaluating this systematic review. First, there is a lack of uniformity in the definition and measure of acculturation. The association between acculturation and ED pathology varied in significance depending on which construct of acculturation was discussed (e.g., acculturation vs. acculturative stress vs. culture change vs. intergenerational conflict). An important future research direction is finding a uniform way to define and measure acculturation. A second limitation is that we did not quantify the size of the relationship between acculturation and ED pathology via a meta-analysis. We chose not to conduct a meta-analysis due to the lack of uniformity in the definitions and measure of acculturation, as we would be unable to draw any meaningful conclusions regarding overall effect sizes. Relatedly, there weren’t enough studies to pursue moderation analysis to understand how differences in the definition/measures of acculturation may impact findings. Instead, we hoped this systematic review would guide future research examining the relationship between acculturation and ED behaviors and cognitions. A third limitation is that most of the included studies comprised undergraduate female students, which limits the generalizability of these findings. The college years are a unique time where undergraduate students may spend a considerable amount of time with their peers and therefore be particularly influenced by mainstream US values. Future research studies should examine these relationships across the lifespan. Relatedly, most of the research on acculturation has been conducted in Hispanic/Latino samples and often using measures only relevant to those specific groups (e.g., the Short Acculturation Scale for Hispanics), further limiting the generalizability of results. Future research should be conducted in other racial and ethnic groups, as their process of acculturation may be different and therefore have distinct associations with eating pathology. Fourth, we did not consider gray literature in order to only include high-quality peer-reviewed articles. However, published literature on the topic is very limited. We were only able to include 22 manuscripts in this review. Future research will need to be conducted on these topics to replicate these findings. Finally, the study was not pre-registered. This was because the first author completed this project as part of her master’s thesis, and she was unsure if she was going to pursue publication when beginning the project.

### Clinical implications

Findings suggest several important relationships between acculturation and its domains with ED behaviors and cognitions. Several intervention practices could reduce risk of ED pathology. For example, direct prevention programs targeting individuals migrating to Western cultures may be beneficial. Specifically, psychoeducation may promote cross-cultural understanding to foster integration, which is the acculturation strategy linked with the most positive outcomes [9, 10]. Additionally, integrating interpersonal effectiveness skills to navigate acculturation gaps that may arise in families may alleviate their overall stress and risk for ED pathology [22]. More broadly, societal advocacy efforts could focus on changing Western values of thinness and appearance.

## Conclusion

Overall, we found that culture change, acculturative stress, and intergenerational conflict were associated with ED behavioral and/or cognitive symptoms. However, the nature of the specific associations differed depending on the specific acculturation constructs and ED cognitions and behaviors measured. In addition, the specific acculturation strategies used were associated with ED pathology, with some strategies contributing to ED pathology but others serving as protective factors. Overall, this review highlights the nuanced relationship between acculturation and eating disorders and the need for more precise definitions of the different domains of acculturation.

## Data Availability

Data are available upon request.
